# Hydro-climatic changes of wetlandscapes across the world

**DOI:** 10.1038/s41598-021-81137-3

**Published:** 2021-02-02

**Authors:** I. Åhlén, G. Vigouroux, G. Destouni, J. Pietroń, N. Ghajarnia, J. Anaya, J. Blanco, S. Borja, S. Chalov, K. P. Chun, N. Clerici, A. Desormeaux, P. Girard, O. Gorelits, A. Hansen, F. Jaramillo, Z. Kalantari, A. Labbaci, L. Licero-Villanueva, J. Livsey, G. Maneas, K. L. McCurley Pisarello, D. Moshir Pahani, S. Palomino-Ángel, R. Price, C. Ricaurte-Villota, L. Fernanda Ricaurte, V. H. Rivera-Monroy, A. Rodriguez, E. Rodriguez, J. Salgado, B. Sannel, S. Seifollahi-Aghmiuni, M. Simard, Y. Sjöberg, P. Terskii, J. Thorslund, D. A. Zamora, J. Jarsjö

**Affiliations:** 1 Department of Physical Geography and Bolin Center for Climate Research, Stockholm University, 10691 Stockholm, Sweden; 2 WSP Sverige AB, Ullevigatan 19, 411 40 Gothenburg, Sweden; 3 Facultad de Ingeniería, Universidad de Medellín, Carrera 87 30-65, 050026 Medellín, Colombia; 4 Facultad de Ciencias Exactas y Naturales, Instituto de Biología, Universidad de Antioquia, Calle 70 No. 52-21, 050010 Medellín, Colombia; 5 Faculty of Geography, Lomonosov Moscow State University, Moscow, Russia 119991; 6 Department of Geography, Hong Kong Baptist University, Hong Kong, SAR China; 7 Department of Biology, Faculty of Natural Sciences and Mathematics, Universidad del Rosario, 13409 Bogotá, DC Colombia; 8 School of Natural Resources and Environment, University of Florida, Gainesville, FL 32603 USA; 9 Centro de Pesquisa do Pantanal and BioScience Institute, Federal University of Mato Grosso, Cuiabá, Mato Grosso Brazil; 10 Zubov State Oceanographic Institute, Moscow, 119034 Russia; 11 Department of Civil, Environmental and Architectural Engineering, University of Kansas, Lawrence, KS 66045 USA; 12 Baltic Sea Centre, 10691 Stockholm, Sweden; 13 Department of Geology, Faculty of Sciences, Ibn Zohr University, Agadir, Morocco; 14 Institute of Botany and Landscape Ecology, University of Greifswald, 17489 Greifswald, Germany; 15 Navarino Environmental Observatory, 24 001 Messinia, Greece; 16 Department of Soil and Water Sciences, University of Florida, Gainesville, FL 32611 USA; 17 Facultad de Ingeniería, Universidad de San Buenaventura, Carrera 56C N° 51–110, 050010 Medellín, Colombia; 18 Department of Earth and Environment, Southeast Environmental Research Center, Florida International University, Miami, FL 33199 USA; 19 Instituto de investigaciones marinas y costeras de Colombia “José Benito Vives de Andreis”—INVEMAR, 470006 Santa Marta, Colombia; 20 Alexander von Humboldt Biological Resources Research Institute, Calle 28 A No. 15-09, Bogotá, DC 70803 Colombia; 21 Department of Oceanography and Coastal Sciences, College of the Coast and Environment, Louisiana State University, Baton Rouge, LA 70803 USA; 22 Civil and Agricultural Engineering Department, Universidad Nacional de Colombia, 11001 Bogotá, Colombia; 23 Departamento de Ciencias Biológicas, Universidad de Los Andes, Cra. 1 No. 18A-12, 111711 Bogotá, Colombia; 24 Facultad de Ingeniería, Universidad Católica de Colombia, Av. Caracas No. 46-72, 111311 Bogotá, Colombia; 25 Jet Propulsion Laboratory, California Institute of Technology, Pasadena, CA USA; 26 Department of Geosciences and Natural Resource Management, University of Copenhagen, Copenhagen, Denmark; 27 Department of Physical Geography, Utrecht University, Utrecht, The Netherlands

**Keywords:** Ecology, Climate sciences, Environmental sciences, Hydrology

## Abstract

Assessments of ecosystem service and function losses of wetlandscapes (i.e., wetlands and their hydrological catchments) suffer from knowledge gaps regarding impacts of ongoing hydro-climatic change. This study investigates hydro-climatic changes during 1976–2015 in 25 wetlandscapes distributed across the world’s tropical, arid, temperate and cold climate zones. Results show that the wetlandscapes were subject to precipitation (P) and temperature (T) changes consistent with mean changes over the world’s land area. However, arid and cold wetlandscapes experienced higher T increases than their respective climate zone. Also, average P decreased in arid and cold wetlandscapes, contrarily to P of arid and cold climate zones, suggesting that these wetlandscapes are located in regions of elevated climate pressures. For most wetlandscapes with available runoff (R) data, the decreases were larger in R than in P, which was attributed to aggravation of climate change impacts by enhanced evapotranspiration losses, e.g. caused by land-use changes.

## Introduction

Globally, wetlands are found in all climate zones. Because region-specific hydro-climatic and geomorphological conditions govern the evolution, prevalence, and characteristics of wetland systems^1, 2^, their ecosystem services and functions may vary between different geographical regions. Peatlands, for instance, hold an essential share of the world’s carbon storage while estuaries and coastal wetlands are important for food provision and biodiversity support. Combined, such various wetland ecosystem services and functions can benefit the society, the environment and the economy of a region. They can therefore be important for reaching multiple Sustainable Developments Goals (SDG), such as those targeting climate and water regulation as well as water purification^3^. The actual consideration of ecosystem services of wetlands additionally depends on the varying perceptions and valuations of wetland systems of different communities and societies. In some parts of the world, wetlands are directly connected to local human survival and societal development, for instance by providing food and contributing to securing drinking water sources. In other parts of the world, wetlands may primarily be valued for services to specific societal sectors, such as improving environmental conditions, e.g., by pollutant retention or maintaining a high biodiversity^4^.

Since wetlands are hydrologically connected to each other and to other landscape elements, such as groundwater, rivers and lakes^5^, they contribute to aggregated impacts on catchment scale hydrological conditions, such as flow-variability damping and regulation^6^. Hence, large-scale functions and ecosystem services of wetlands, and how they can be impacted by climate change, should be studied as aggregated units instead of individually. A way of approaching this, is to take on a wetlandscape perspective^7, 8^, which considers the connected landscape-wetland system defined by the wetlands’ aggregated hydrological catchments. Methodologically, such a perspective is necessary for quantifying (changes in) water balances, and making theoretically sound projections for runoff and water discharges, which are closely related to the evolution of many wetland ecosystem services.

Although wetlands play a role in the climate system, not least as carbon sinks (e.g., saltwater wetlands) or sources of carbon emissions, e.g., high altitude wetlands^9^, they are themselves vulnerable to ongoing hydro-climatic changes in their catchments^10^. In addition, wetlands are vulnerable to spatio-temporally overlapping effects of land-use changes, which can have considerable impacts on the water cycling through them^11^. Such land-use changes can be related to ditching of wetland areas for conversion into arable land, agricultural intensification including irrigation expansion, as well as urban and industrial development. More generally, whereas many regions of the world are subject to water cycle changes that e.g. impact runoff, there is considerable variability in both magnitude and direction of change, as well as in the drivers of change^11^. Due to the complexity, detailed assessments are frequently needed to understand cause-and-effect. There are for instance numerous examples of regions exposed to precipitation increases, which simultaneously, and counter-intuitively, experience runoff decreases because of various overlapping effects^12^ (e.g. of temperature increases and agricultural development). For understanding net results, one may also need to account for the fact that the impact of overlapping change drivers, due to interaction effects, can differ from what would be expected from the sum of the individual impacts^13^ (of one driver at a time). Taken together, the above-described, complex processes have potential implications for biodiversity^14^, carbon sequestration^9^, water availability^15^ and water quality^16^ of wetlandscapes, depending on the ambient conditions.

In response to major historic and present wetland deterioration, efforts to preserve and restore wetlands and their ecosystem services are now increasing in different parts of the world^10^. Wetland restoration is frequently seen as important in using nature-based solutions for addressing regional environmental challenges, not least as they can store water and improve water quality. Despite the relevance of large-scale wetlandscape interactions with climate change and other change drivers, investigations of wetlandscapes are less considered in the scientific literature compared to local changes of individual wetlands^7^. As a result, the knowledge on both current and possible future interactions of wetlandscapes with climate change and other change drivers is limited, which in turn limits possibilities of science-based wetland management decisions.

In this study, we consider wetlandscape data from the global WetCID dataset^17^ and analyze long-term hydro-climatic data (over the study period of 1976–2015) for 25 different wetlandscapes, of which 9 have additional stream discharge (Q) data. Our working hypothesis is that, compared to hydro-climatic characteristics of the world’s different climate zones, there may be systematic differences in parameter values, trends and variability for less studied wetlandscapes within the climate zones. A possible reason would be that wetlandscapes are not randomly distributed across landscapes since their occurrence require certain hydro-climatic and geomorphological conditions to be fulfilled. Furthermore, we hypothesize that change drivers may differ considerably between different wetlandscapes with important implications for ecosystem services, as previously seen for other (non-wetlandscape) regions.

Hence, this paper addresses the following main research questions: (i) For which wetlandscapes have the corresponding changes in driving atmospheric hydro-climatic variables temperature (T) and precipitation (P) been most significant and how do these changes differ among wetlandscapes? (ii) Are the magnitudes of change in T and P in the studied wetlandscapes consistent with the average T and P changes over their respective climate zones (i.e., in which each wetlandscape is located)? (iii) How do changes in water runoff (R, i.e., measured stream discharge, Q, normalized with contributing wetlandscape catchment) through the wetlandscapes relate to the atmospheric T and P changes? For instance, are the R changes consistent with and more or less fully explained by T and P changes, or are there other change drivers, such as various human land- and water-use developments in the wetlandscapes themselves, likely important contributing factors for the R changes?

## Results

**Figure 1:**
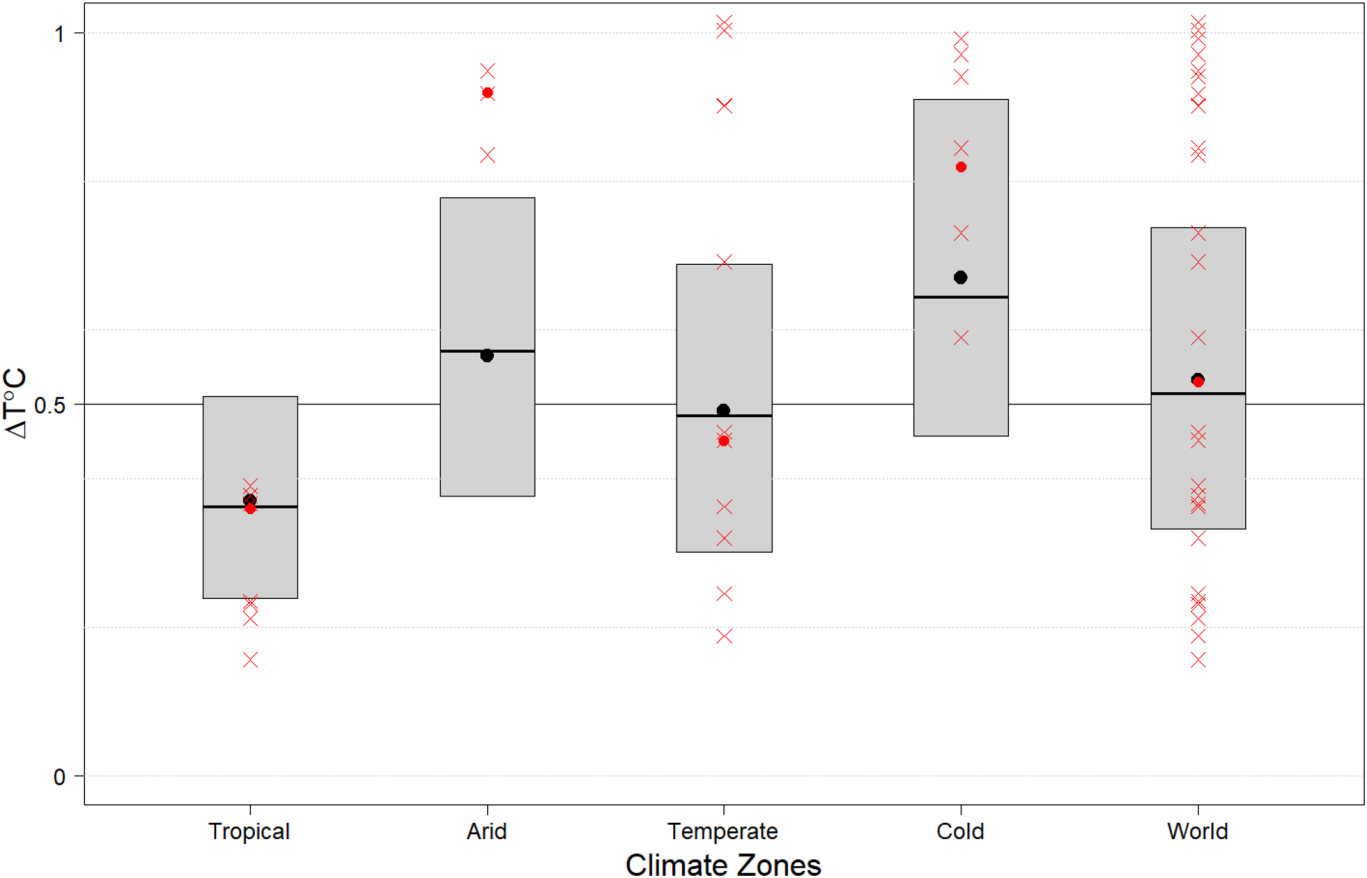
Change in temperature (ΔT°C). Boxplot (without whisker) showing the ΔT°C for each climate zone between the periods of 1976–1995 and 1996–2015. The ΔT°C for each wetlandscape is shown as redcrosses. Mean ΔT°C for the climate zones are shown as black points, while the red points show the mean ΔT°C for the wetlandscapes. For detailed boxplots with whiskers, see Supplementary Material.

On the continental scale, all climate zones show increasing temperatures (positive Δ*T-*values) between the period 1 (1976–1995) and period 2 (1996–2015) (Fig. 1), although to different extents. The largest ΔT is seen for the arid and cold climate zones, as median ΔT (Fig. 1, black line) and mean ΔT (Fig. 1, black point) for those climate zones exceed 0.5 °C (world median value), in contrast to the tropical and temperate climate zones. All of the wetlandscape sites also show statistically significant (p < 0.05) positive ΔT (Fig. 1, red crosses; Table S2). However, the mean ΔT for the arid wetlandscapes (Fig. 1, red point) is considerably higher (0.36 °C) than the mean ΔT of both the arid climate zone as a whole and the share of the arid climate zone located within the northern hemisphere (Fig. 1, black point and Fig. S6). This is also true for the wetlandscapes within the cold climate zone (where the corresponding difference is 0.15 °C).

**Figure 2:**
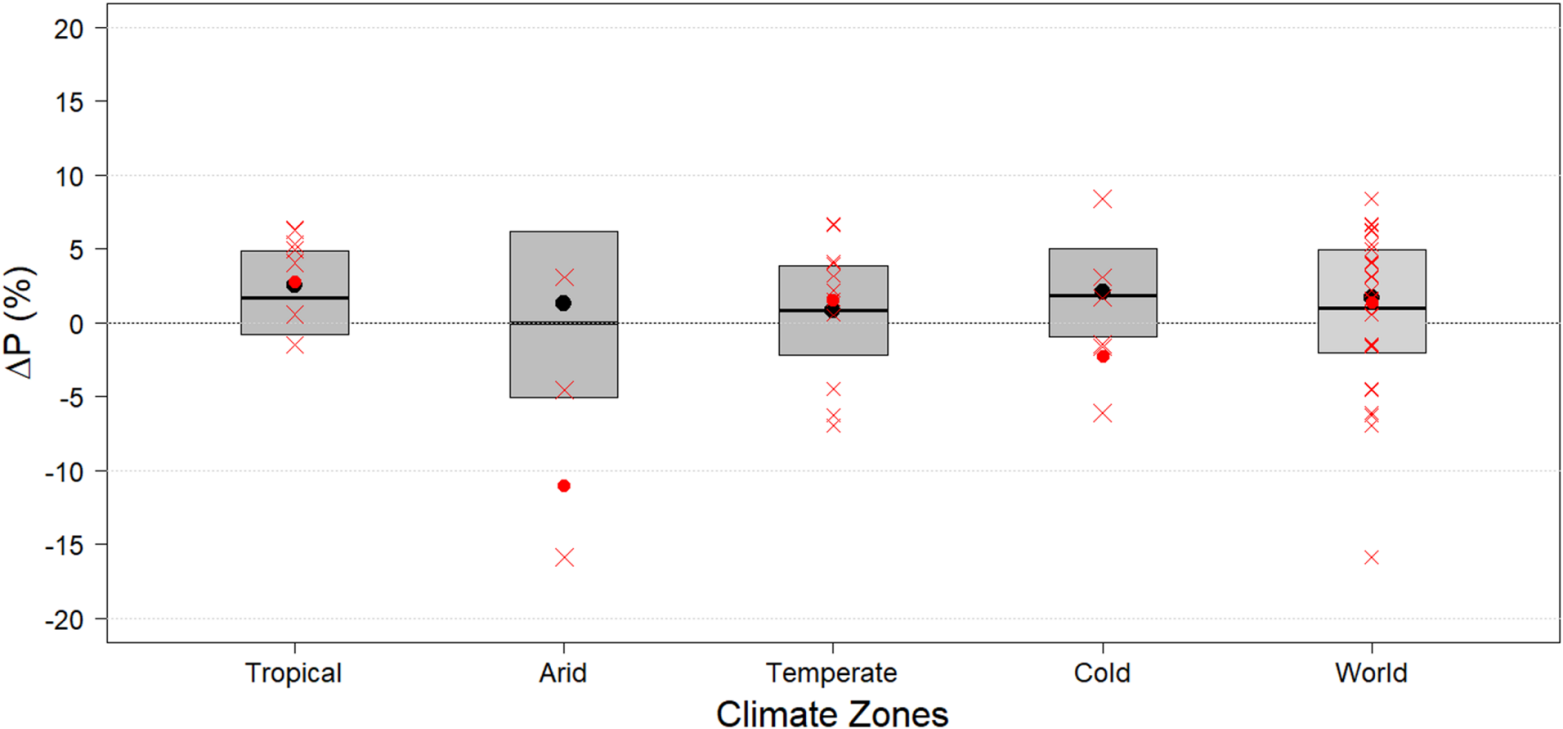
Relative change in precipitation (ΔP%). Boxplots (without whiskers) show the relative ΔP in percentage for each climate zone for the periods between 1976–1995 and 1996–2015. The relative ΔP for each wetlandscape is presented as red crosses. Mean ΔP% for the climate zones are shown as black points, while the red points show the mean ΔP% for the wetlandscapes. For detailed boxplots with whiskers, see Supplementary Information.

The mean ΔP (black points) is positive for all climate zones between period 1 and period 2 (ΔP > 0; Fig. 2), implying increased mean P. The median ΔP (black lines) is also positive, except for the arid climate zone where it is unchanged. The largest relative increase in mean P is seen for the tropical climate zone (Fig. 2). This increase becomes even more pronounced when expressed in absolute terms (Fig. S3 of the Supplementary Information), since precipitation is generally high in the tropics. The smallest relative change in mean P is seen for the temperate climate zone (Fig. 2), whereas the smallest absolute change is seen for the arid climate zone, due to low precipitation. Moreover, although the spread expressed as the interquartile range of ΔP within the tropical climate zone is the largest of all climate zones in absolute terms (Fig. S3, height of grey box), the opposite is true in relative terms (Fig. 2). Conversely, the interquartile range of ΔP within the arid climate zone is among the lowest in absolute terms (Fig. S3; together with the cold climate zone), but highest in relative terms (Fig. 2).

The mean precipitation increases for tropical and temperate wetlandscapes (Fig. 2; red points) are practically identical to the precipitation increases in their respective continental-scale climate zone (Fig. 2, black points). In contrast, the arid and cold wetlandscapes have on average been exposed to precipitation decreases, whereas their corresponding climate zones as well as the share of their climate zones located within the northern hemisphere on average have experienced precipitation increases (Figs. 2, S8). Furthermore, a common characteristic of the arid, temperate and cold wetlandscapes is that the variability is considerable between individual wetlandscapes (Fig. 2; red crosses). Hence deviations from the average trend of the climate zone is common (e.g., three out of six cold wetlandscapes are subject to precipitation increases, even though the ensemble average shows a precipitation decrease). There is a non-negligible variability also among the tropical wetlandscapes, however, the vast majority (6 of 7 wetlandscapes) of those are subject to precipitation increases although not statistically significant (p > 0.05; Table S2).

**Figure 3:**
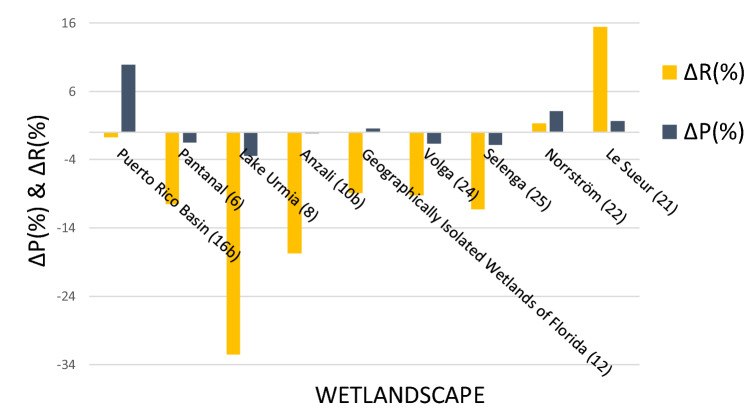
Relative change of precipitation and runoff for wetlandscapes with available discharge data. The nine wetlandscapes are here presented with name and corresponding ID number. The change in precipitation and discharge for the nine wetlandscapes corresponds to the different time periods in Table 1.

Considering relative changes over time, a majority (6 of 9) of the wetlandscapes with available Q data showed considerable decreases in R (− 10% < ΔR <  − 35%; yellow bars in Fig. 3), two of which were statistically significant (p < 0.05; Pantanal and Anzali, Table S2). This is despite the fact that corresponding P typically either show just a modest decrease (ΔP of minus a few %; blue bars in Fig. 3), or even small increases (ΔP of plus a few %). For many (four of nine) of the wetlandscapes, R additionally show larger absolute changes than P (Figs. 3, S4), despite the fact that R is lower than P (on average 34% of P). The three wetlandscapes not showing distinct R decreases are two cold and one tropical wetlandscape, namely the Norrström drainage basin, Sweden (ID 22; Fig. 3), Le Sueur, USA (ID 21), and the Puerto Rico basin, Colombia (ID 16b). For Norrström, the changes in both R and P are negligible, whereas Le Sueur exhibits considerable increase in R (ΔR ≈ 15%) despite the fact that the change in P is insignificant. Finally, the tropical Puerto Rico basin shows unchanged R despite being subject to a considerable increase in P (ΔP ≈ 10%).

**Figure 4:**
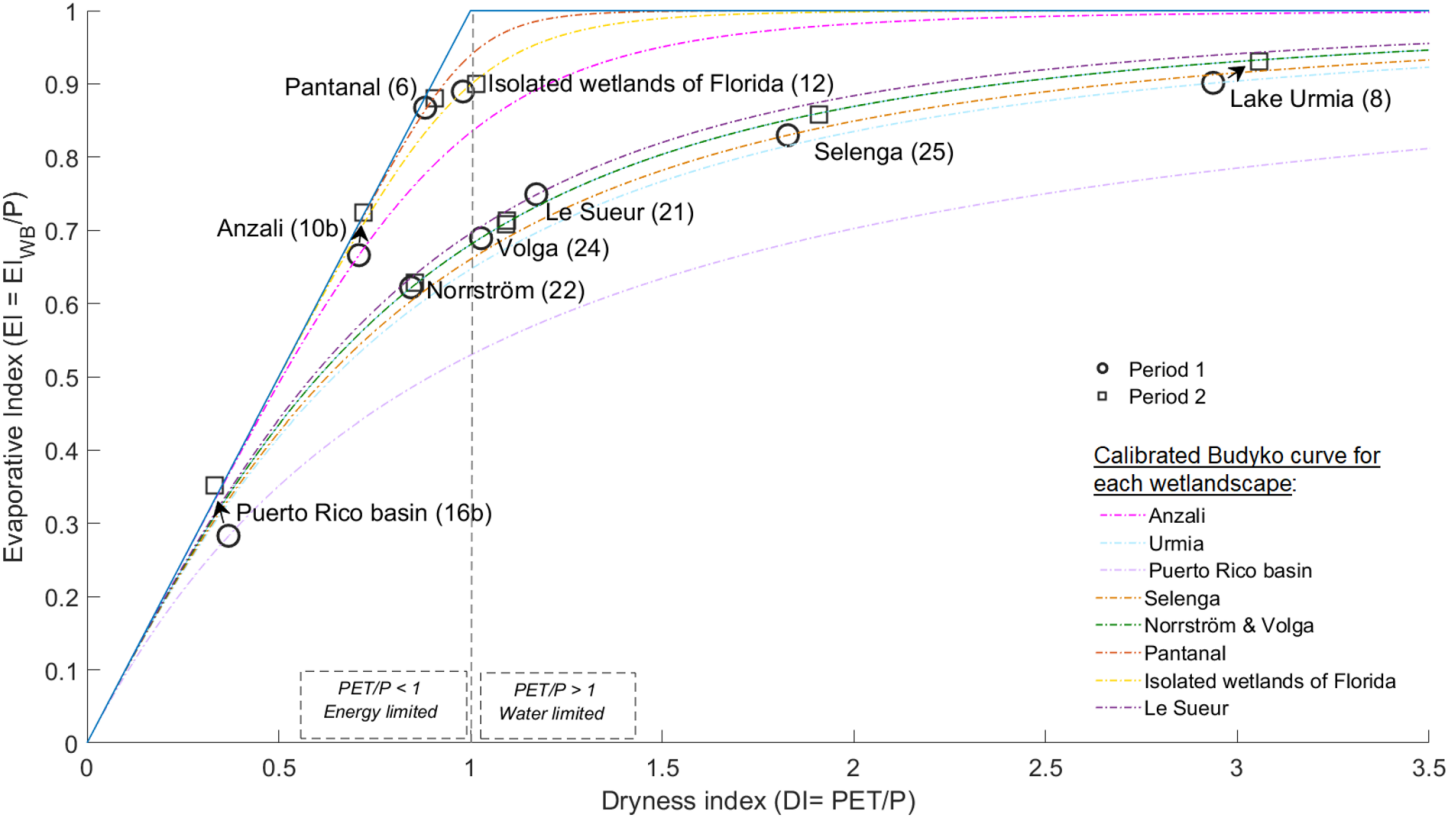
Wetlandscape hydro-climatical conditions presented in the Budyko space. Relation between the evaporative and dryness index for the first (circle point) and the second period (box point) of the studied time period for all the different wetlandscape with available Q data. All wetlandscapes are represented with a calibrated Budyko curve representing their region specific climatic conditions.

The evapotranspiration losses of water for some of the studied wetlandscapes are limited by the availability of (solar) energy as reflected in the Budyko framework in Fig. 4 (left part; DI < 1). The Puerto Rico basin is the most energy-constrained of all studied wetlandscapes, with a DI of around 0.3. In contrast, the evapotranspiration losses in other wetlandscapes are limited by the availability of (precipitation) water (Fig. 4, right part; DI > 1). The Lake Urmia basin is the most extreme example showing DI of around 3. That basin, together with the Selenga River basin, are the two driest wetlandscapes, and were so already during the first (reference) period (Fig. 4, circle points). Despite their initial dryness, these two basins are the ones experiencing the largest shifts along the x-axis to even dryer conditions from the first to the second period (Fig. 4, box points).

**Figure 5:**
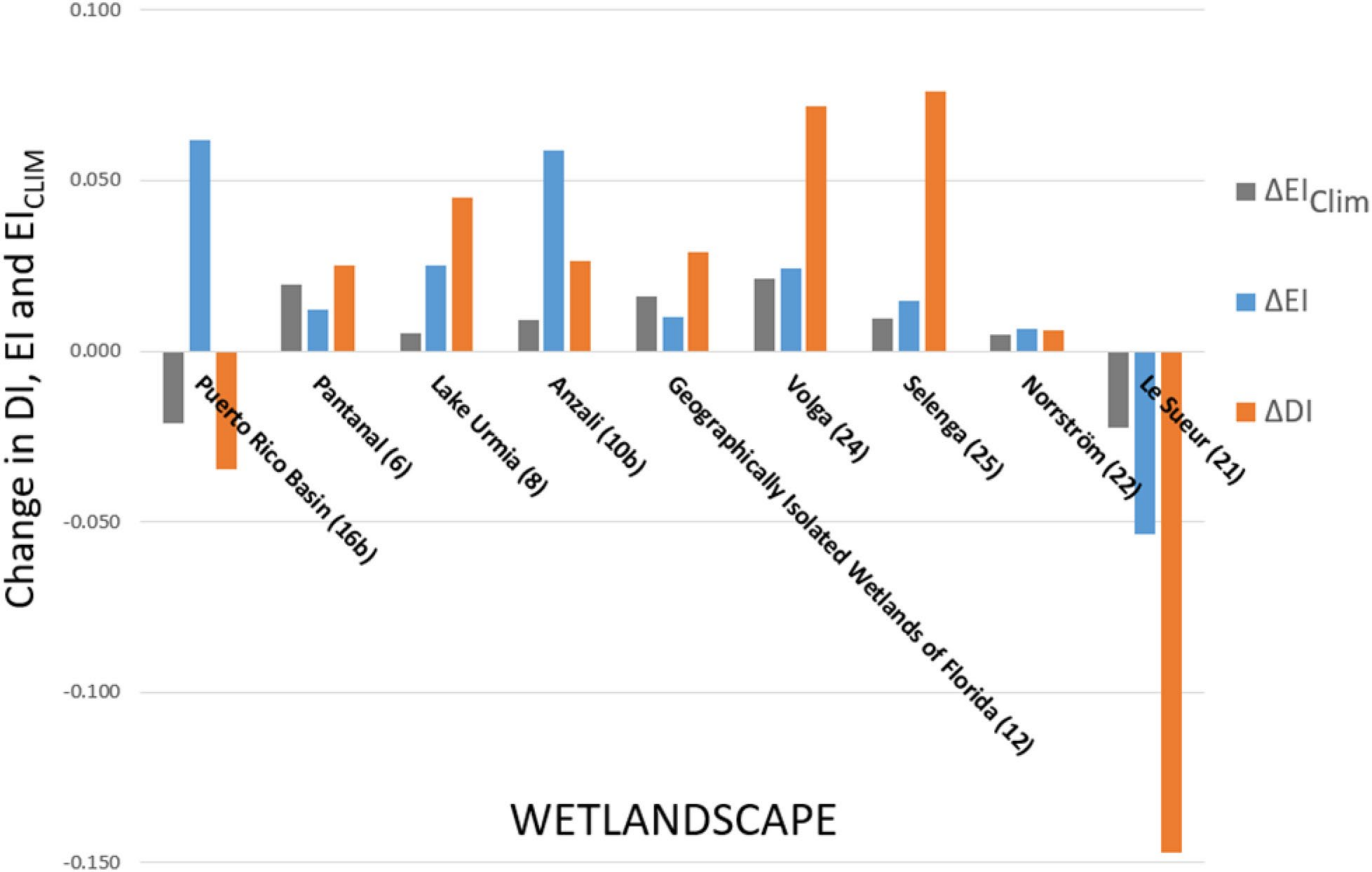
Change in evaporative and dryness index for wetlandscapes with available discharge data. Change in the evaporative indices (ΔEI) are represented as blue bars and as grey bars for ΔEI_Clim_. Change in dryness index (ΔDI) is shown as orange bars. The changes in evaporative and dryness indices corresponds to the time period of the discharge data for these wetlandscapes (Table 1).

Figure 5 summarizes how DI and EI have changed between period 1 and period 2, and shows that most wetlandscapes (7 of 9) have been subject to increase in DI (Fig. 5; orange bars), which has also been larger than the increase in EI (Fig. 5; blue bars). Only for the cold wetlandscape of Le Sueur, the DI has decreased, as has also EI (Fig. 5), where the later change implies increase in the runoff coefficient, defined as runoff relative to precipitation (R/P). The tropical Puerto Rico basin also exhibits a decrease in DI, accompanied in this case by a considerable increase in EI (Fig. 5), which implies a correspondingly large decrease in R/P. This increased EI cannot be explained by changes in climatic variables (T and P), as shown by the fact that EI_Clim_ (Fig. 5; grey bar) has decreased. Moreover, the Anzali and Lake Urmia wetlandscapes have experienced a similarly large increase in EI (and decrease in R/P), however with EI_Clim_ showing only a relatively small increase. For most wetlandscapes (Anzali and Puerto Rico basin included), atmospheric climate change in T and P has only partly (or insignificantly) contributed to observed EI change ($\left| {{\text{EI}}_{{{\text{Clim}}}} } \right| \le \left| {{\text{EI}}} \right|$ in 7 of 9 wetlandscapes). In a few cases, like Pantanal and Volga, the EI changes seem consistent with corresponding climatic changes EI_Clim_ between the periods 1976–1995 and 1996–2015 (Fig. 5). Furthermore, the EI changes for the Puerto Rico and Anzali wetlandscapes, have made these strictly energy limited (as reflected by results for the second period in the Budyko plot; Fig. 4, box points). This implies that the evaporative index has reached its maximum possible value, which essentially prohibits further increases unless the basin starts to move towards a dryer state.

## Discussion

At a global scale, our results show that the mean change in P and T for the studied 25 wetlandscapes are similar to the changes in T and P of the world’s continents. Even so, we find that the conditions in many wetlandscapes differ from the world average ones, due to a considerable variability in hydro-climatic variables and other conditions between wetlandscapes. We show that some of this variability between the considered wetlandscapes is explained by systematic differences in hydro-climatic conditions (and trends) between the main climate zones of the world. However, whereas the P and T changes within the tropical and temperate climate zones are representative for the average changes of the 16 wetlandscapes located within these climate zones, this is not the case for the 9 arid and cold wetlandscapes. The latter show pronounced decreases in P, and relatively high increases in T, which did not reflect average changes within these zones. This has driven the considered arid and cold wetlandscapes to much drier states than what would be expected from the average trends within the climate zones. A possible reason for this is that (non-random) locations of arid and cold wetlandscapes within the landscape may correlate with locations subject to high climate pressures compared to climate zone averages (due to for instance topography and distance from coastal areas). Consequently, it may not be appropriate to use average changes in T and P of the climate zone as a proxy for understanding these variables in wetlandscapes, and particularly not for arid and cold wetlandscapes. Whereas the overall generality of this finding remains to be further investigated, we note that in any case, the 9 studied arid and cold wetlandscapes (found to be under high change pressures) include major wetlandscapes of the world, in terms of areal extent and population density, such as Lake Urmia (Iran), Volga (Russia) and Selenga (Russia).

Most of the wetlandscapes show considerable decreases in R and consequently decreases in water flows through their wetlands in the past couple of decades, despite the fact that they were typically subject to relatively small changes in P. This implies that evapotranspiration over the wetlandscapes and associated runoff coefficients must have changed considerably^12,18^. Although climate change can be associated with increased T, increased evapotranspiration and increased dryness for all of the considered wetlandscapes, our results showed that such climate-induced dryness (i.e., dryness driven by changes in P and T) could only explain a part of the observed R decreases and changes in evaporative loss (EI). The remaining, unexplained part is most likely related to the considerable changes in land-use and other anthropogenic pressures that most wetlandscapes have undergone in recent decades. This means that regional observations of ΔP and ΔT are not suitable as predictors for the evolution of the wetlandscape ΔR, which is a key variable for many ecosystem services^2^. It emphasizes the need for using discharge measurements in hydro-climatic assessments of wetlandscapes, and for testing and verifying hypotheses regarding the impacts of land use change on wetlandscape ecosystem services and functioning. For instance, in the case of the wetlandscape of Ciénaga Grande de Santa Marta, irrigation, damming and dispatching of water from the wetlands have been hypothesized to be important factors behind increasing salinity rates and massive mangrove degradation. However, conclusive results are lacking because discharge data remains very limited^15^.

Analyses of calibrated Budyko curves showed that the wetlandscapes of the Puerto Rico basin (Colombia) and Anzali (Iran) where not subject to pressures from the investigated climatic variables, which made the impacts of land-use change and similar anthropogenic pressures particularly clear. This is because these wetlandscapes have undergone considerable changes in the EI, despite a lack of impact from climatic variables (Fig. 4). In the Puerto Rico basin, for instance, the ΔT increased with only 0.12 °C between 1976–1995 and 1996–2015. The increases in EI of the Puerto Rico basin is nonetheless consistent with increasing pressures from grazing and considerable changes in vegetation cover^19,20^, which could explain increases in evapotranspiration and reduced runoff formation. In the Anzali basin, similar effects can be seen as a result of damming and intensified agriculture in recent decades^21^. Both basins have been subject to changes to the extent that their evapotranspiration is now strictly energy limited. The resulting changes in hydro-climatic conditions for Anzali have lead to increased salinity levels affecting the water quality^21^ within the Anzali wetlandscape and reduced water availability within the Puerto Rico basin^20^. Consequently, these environmental changes are putting considerable pressures on biodiversity support, water supply and food production in these regions^4^.

Similar conclusions can be drawn for hydro-climatic changes of the Lake Urmia wetlandscape (Iran), although the increase in EI is not as large as it is for e.g., the Anzali wetlandscape. Hence, the observed rather small change in EI_Clim_ in comparison to the change in EI indicates a smaller climate effect with stronger effects of anthropogenic pressures. These results are in line with previous studies on Lake Urmia showing that decreases in R and increases in EI are mainly due to the mega-scale agricultural development including irrigation expansion in the past decades and explains the dramatic shrinkage of Lake Urmia^22^. The development is furthermore similar to the disastrous development of the Aral Sea, which experienced a transition that started to accelerate already in the 1990’s, leading to water volume losses of more than 90%, salinities above 100 g·L^−1^, and a total loss of water ecosystems. Apart from impacts on fisheries, which now exist only to small extent due to construction of freshwater reservoirs, soil salinization severely impacts agriculture and food production in the basin. This disaster is almost entirely due to mega-scale agricultural development, including cotton production^23,24^ in the drainage basin of the Aral Sea. For the Lake Urmia basin a further decrease in P would aggravate the conditions of rivers that are already running dry most time of the year and surface water bodies that are drastically shrinking. The degradation of Lake Urmia, including severe salinization and ecosystem collapse^25^, has eradicated its water ecosystem and along with it the region’s fisheries, and agriculture is under pressures from groundwater salinization and wind-blown dust and salt.

Furthermore, the Selenga basin has been subject to T increases well above the global average, and the largest increase in DI among all studied wetlandscapes, which can be associated with widespread permafrost thaw^26^. Hence, even though ΔP over the Selenga basin has shown the largest decrease, in relative terms, of all cold wetlandscapes considered in the present study, the relative R decrease has been even more pronounced due to increased evaporative water losses. This is particularly the case for the summer season, which is when the highest decrease in precipitation occurred while the evapotranspiration is still high. The fact that the largest hydro-climatic changes of the Selenga basin have occurred during the main growing season has adversely impacted water-sensitive ecosystems of the Selenga delta, including the now-endangered, famous Baikal (Siberian) sturgeon from whose roe caviar is produced. Concerns have furthermore been raised regarding geomorphological changes of the delta wetlands^27^ impacting biodiversity and water quality of the coastal waters of Lake Baikal.

Le Sueur is our only wetlandscape example where both EI and DI has decreased. While the decrease in DI is related to climatic factors (increased P and modest T increase), the climatic factors could however only partly explain the observed decrease in the EI. Le Sueur basin agriculture has shifted away from hay and small grains, which made up a third of crops in the 1970s, to primarily corn and soybean production by 2015^28^. In addition to the industrialization of agriculture in the basin, the extent of artificial subsurface drainage systems increased, which may contribute to increases in runoff coefficients and decreases in EI. This has been accompanied by losses of wetlands from the wetlandscape^28,29^ impacting surface water availability and water quality^4^.

As shown by the large variability in hydro-climatic conditions of the here considered wetlandscapes, their drivers of change can generally not be accurately understood from regional averages, such as the hydro-climatic characteristics of the climate zone they are located in. Consideration of local hydro-climatic data are in most cases required for the 25 wetlandscapes of the present study. However, for precipitation, even local trends are unhelpful for predicting runoff changes in wetlandscapes, since correlations between precipitation trends and runoff trends are weak or non-existent. Consequently, for reducing otherwise large uncertainties in assessments of hydro-climatic and geomorphologic changes of wetlandscapes, there is a need for local measurement data on discharge and water levels, which often are unavailable. Such hydrological data should therefore be given greater importance in the development of environmental datasets on the wetlandscape scale. This is also important for developing sustainable management practices for wetlandscapes to support ecosystem services such as water provision, water quality, biodiversity support, and project possible geomorphological changes of wetlands^27,30–32^.

## Methods

**Figure 6:**
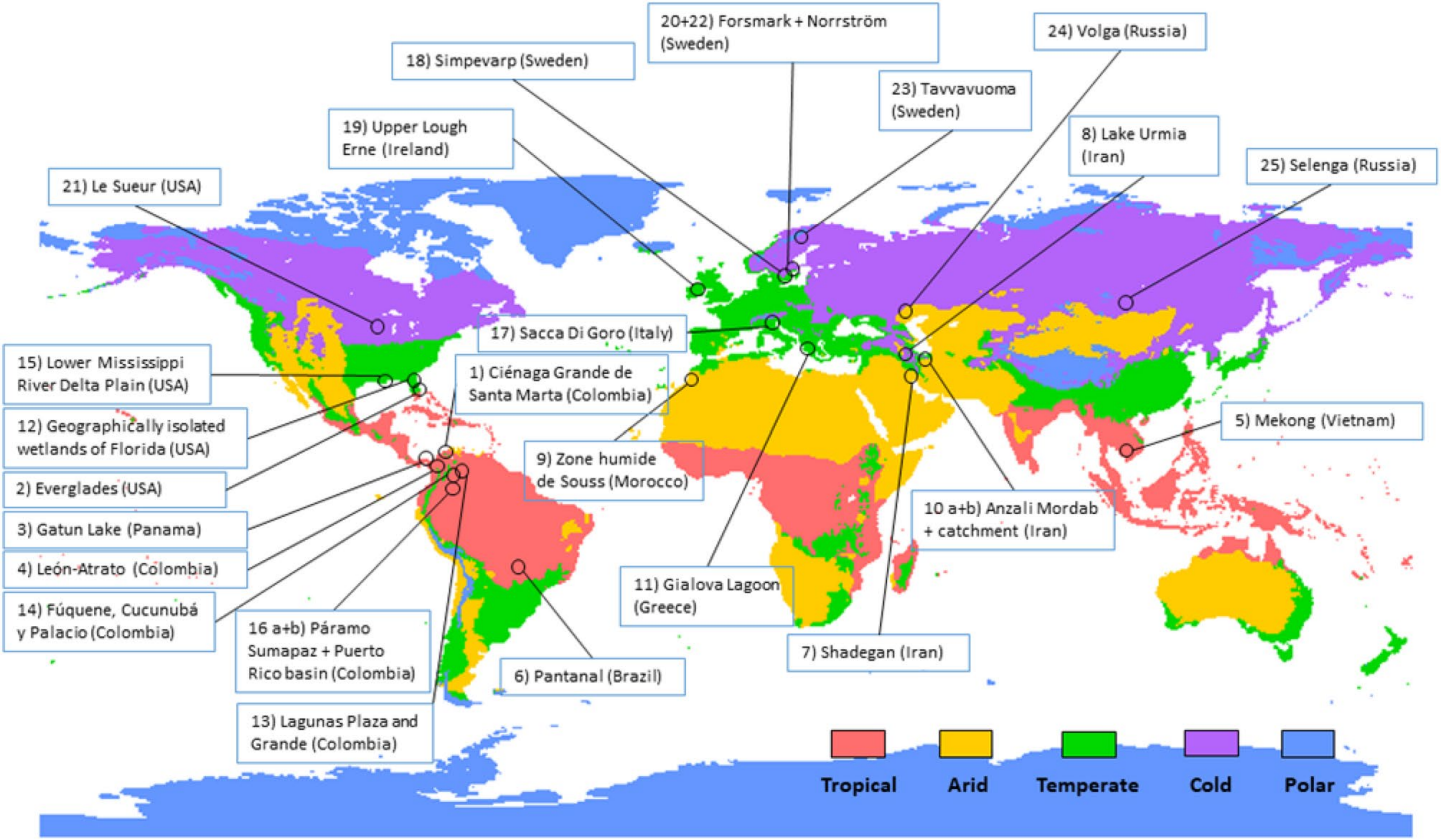
Map over the 25 studied wetlandscapes. Distribution of the 25 GWEN wetlandscapes included in this study over four of the five main climate zones (Tropical, Arid, Temperate and Cold), classified according to the Köppen-Geiger climate classification system (Kottek et al., 2006). The wetlandscape site numbers correspond to the ID numbering in Table 1. This map was created by the authors using QGIS 3.12.2 (https://www.qgis.org/en/site/forusers/visualchangelog312/index.html).

In order to quantify changes in hydro-climatic conditions of the studied wetlandscapes and understand whether or not these changes are related to their geographical location, we considered T and P data over all continents (except for the polar regions). The data were grouped into four main climate zones (tropical, arid, temperate, cold) according to the updated Köppen–Gieger classification system^33^. We then analyzed hydro-climatic trends of the climate zones, as well as of the 25 wetlandscapes located within these zones (Fig. 6). The wetlandscapes were retrieved from the WetCID database^17,34^ (https://doi.org/10.1594/PANGAEA.907398), which is a unique compilation of wetlandscape data (geographical, hydro-climatic and land-use data on the wetlands including their entire hydrological catchments) distributed across four climate zones, initiated by wetland scientists of the Global Wetland Ecohydrological Network (GWEN) (www.gwennetwork.se). The compilation encompasses consistently formatted, peer-reviewed data on well-characterized wetlandscapes of high scientific interest^34^. No wetlandscape data were available for the Polar zone (Fig. 6) in the WetCID database.

We retrieved the longest time series possible without data gaps of T and P from the monthly CRU TS v4.03 dataset^35^ (0.5° resolution and based on direct measurements; see supplementary information for details) which resulted in the analysis of 40 years of T and P data over the period of 1976–2015. As such, the analysis of hydro-climatic (specifically: T and P) changes for the wetlandscapes and climate zones were evaluated based on differences between two 20-year long periods, 1976–1995 (hereafter denoted period 1) and 1996–2015 (denoted period 2). These periods overlapped with the slightly shorter periods during which the discharge (Q) data were also available. In total, Q data were available for nine of the wetlandscapes (Table 1, last column). Similar to the T and P data analyses, we divided the Q datasets into two periods of equal length, from which differences in Q were evaluated. For the wetlandscapes that had Q data, we used the data to calculate R. In turn, R was used to estimate the water balance component of annual evapotranspiration (ET_WB_) as ET_WB_ ≈ *P*-*R*, considering that long-term change in storage would be approximately equal to zero^18^. Additionally, we retrieved Potential Evapotranspiration (PET) from the CRU TS v4.03 dataset.

**Table 1:** Data overview of the studied wetlandscapes.

Climate zone	ID	Wetlandscape	Coordinates (decimal degrees)		Area (km^2^)	∆T (°C)	∆P (mm/year)	Time period of *Q* data (n.a. = not applicable)
			Latitude	Longitude				
Tropical	1	Ciénaga Grande de Santa Marta (Colombia)	10.54	− 74.87	267,000	0.23	121.75	n.a.
	2	Everglades (USA)	25.34	80.93	56,557	0.38	66.79	n.a.
	3	Gatun Lake (Panama)	9.26	79.92	2937	0.23	14.76	n.a.
	4	León-Atrato (Colombia)	7.94	− 76.75	2334	0.21	163.26	n.a.
	5	Mekong (Vietnam)	9.82	106.53	803,000	0.39	59.7	n.a.
	16b	Puerto Rico basin (Colombia)	2.95	73.21	5960	0.12	288	1980–2014
	6	Pantanal (Brasil)	17.26	57.45	611,000	0.37	− 23.7	1976–2015
Arid	7	Shadegan (Iran)	30.5	48.75	13,553	0.92	− 9.46	n.a.
	8	Lake Urmia (Iran)	37.5	45.5	51,825	0.95	− 60.26	1976–2015
						0.7	− 12.92	1986–2015
	9	Zone humide de Souss (Morocco)	30.36	− 9.5	16,160	0.84	7.98	n.a.
Temperature	10a	AnzaliMordaban)	37.42	49.47	3830	1	− 28.69	n.a.
	10b	Anzali catchment (Iran)			2365	0.66	− 1.87	1986–2014
	11	Gialova Lagoon (Greece)	36.96	21.67	180	0.69	26.26	n.a.
	12	GeographicallyIsolated Wetlands of Florida (USA)	29.47	− 81.69	7288	0.36	7.03	1976–2015
	13	Laguna Plaza and Grande (Colombia)	6.45	− 72.38	15	0.32	28.07	n.a.
	14	Fúquene, Cucunubá y Palacio (Colombia)	5.5	− 73.78	1204	0.25	59.02	n.a.
	15	Lower Mississippi River delta plain (USA)	31.07	91.58	3,310,000	0.45	12.43	n.a.
	16a	Páramo Sumapaz (Colombia)	3.74	− 73.83	2217	0.19	121.5	n.a.
	17	Sacca Di Goro (Italy)	44.09	12.34	2294	0.9	− 31.51	n.a.
	18	Simpevarp (Sweden)	57.43	16.58	233	0.9	25.92	n.a.
	19	Upper Lough Erne (Ireland)	54.32	7.63	3410	0.46	36.61	n.a.
Cold	20	Forsmark (Sweden)	60.38	18.2	402	0.97	− 7.8	n.a.
	21	Le Sueur (USA)	44.29	93.26	2880	0.59	13.48	1976–2015
	22	Norrström (Sweden)	59.32	17.87	22,654	0.94	18.93	1976–2015
	23	Tawavuoma (Sweden)	65.951	24.04	846	0.99	49.16	n.a
	24	Volga (Russia)	46.76	47.8	1,396,930	0.84	− 9.74	1976–2015
	25	Selenga (Russia)	52.15	106.57	460,000	0.73	− 22.27	1976–2015
						1.15	− 6.5	1976–2009

Table showing the 25 studied wetlandscapes and their corresponding climate zone as well as their ID number, coordinates, name and size. In addition, the change in average temperature and average precipitation (∆T and ∆P) between the first half and the second half the studied time period(s) (1976–2015, or as given in the last column) is presented, as well as the time period of Q data availability.

ET_WB_ was used to estimate the evaporative index $\left( {{\text{EI}} = \frac{{ET_{WB} }}{P}} \right)$, which was compared to the dryness index $\left( {{\text{DI}} = \frac{PET}{P}} \right)$ according to the Budyko framework^36^, for all nine wetlandscapes with Q data. Both the evaporative index and the dryness index were calculated for two time periods of equal length, corresponding to the first and second half of the entire time period of the wetlandscape’s Q data. The EI and DI for period 1 was then compared to the EI and DI of period 2, plotted in the Budyko space in order to interpret changes in hydro-climatological conditions for each of the nine wetlandscapes. More specifically, the Budyko framework provided tools for understanding long-term water and energy constraints/limitations^37^ of the wetlandscapes. To account for differences in physical characteristics (such as soil type, vegetation cover and topography) of each wetlandscape, an adapted version of the Budyko formulation developed by Yang et al.^38^, and later synthesized by Zhang et al.^39^, was used to estimate a specific relation between EI and DI adapted to each wetlandscape according to:

1 $$EI_{Clim} = \left( {1 + \left( {DI} \right)^{n} } \right)^{{\frac{ - 1}{n}}} ,$$

where EI_Clim_ is the evaporative index as derived from the climatic model of Yang et al.^38^, DI is the dryness index and *n* represents specific physical characteristics of each wetlandscape. This adapted Budyko formulation enables an estimation of *n* which is back-calculated from known DI and EI (based on ET_WB_) for period 1 and gives a calibrated Budyko curve for each wetlandscape. A theoretical EI_Clim_ for period 2, which accounts only for differences in climate parameters between the periods but neglects potential changes in *n* (e.g. driven by land-use change) was then calculated for each of the wetlandscapes based on Eq. (1) with DI from period 2 and *n* kept unchanged. We also calculated the corresponding changes in EI (based on ET_WB_), EI_Clim_ and DI between the first and the second periods (ΔEI, ΔEI_Clim_ and ΔDI) for each of the nine wetlandscapes with Q data. This comparison was made to distinguish between changes in evaporative water losses (EI) that essentially can be explained by climate drivers (as reflected by ΔEI_Clim_) and unexplained changes including possible impacts of land use change (as reflected by the difference between ΔEI and ΔEI_Clim_)^18^.

For calculation of T and P trends representative of each of the 25 wetlandscapes, we departed from the definition of a wetlandscape as the union of the wetlands’ hydrological catchments. For high altitude wetlands, the wetlandscapes were defined by the regional headwater zones. The resulting areas of the considered wetlandscapes are shown in Table 1, along with their locations, climate zones and ID numbers, which we refer to in the results section. More detailed information regarding e.g. wetland type and wetland area coverage of the here studied 25 wetlandscapes can be found in Supplementary Material ST2 and through the WetCID dataset^17^. As mentioned above, monthly Q data was additionally available for nine wetlandscapes. In most cases, the Q measurement station was located at the (hydrological) outlet of the wetlandscapes, such that the catchment defined by the discharge station coincided with our delineated wetlandscape area. This was not the case for the wetlandscapes with IDs 10 and 16. In these cases we performed our analyses both for the wetland areas (IDs ending with the letter *a* in Table 1) and for the catchment areas of the available Q measurement station (IDs ending with the letter *b* in Table 1).

For calculation of T and P trends representative of the main Köppen–Gieger climate zones, the gridded CRU raster data were resampled to match the resolution of the gridded data (5′, or 0.0833° resolution) over the climate zones, obtained from Kottek et al.^33^. T and P data were extracted from each of the climate zones. For consistency, if coastal areas of some of the considered layers were represented by “no data” (due to difference in resolution) these areas were excluded from the analysis. Since the input data coordinates were given according to the World Geodetic System (WGS84), which is a non-equal area coordinate system, we had to consider the variation in cell areas with the latitude in the CRU raster. As such, weighted values for all the raster cells, depending on their latitudinal distances from the equator were used. The cells adjacent to the equator were assigned a weight (*w*) value 1.0. The *w* values decreased gradually with an interval of 5′ down to a value of 7.36 × 10^–5^ for the cells adjacent to the poles (north and south) following the principles of Wen et al.^40^.

Specifically, raster layers of ∆T (°C) and ∆P (mm/year) were calculated for each pixel *i* as the difference in the period-averaged $\overline{T}$ and $\overline{P}$ of pixel *i* between the periods 2 and 1, according to:

2 $$\Delta T_{i} = \overline{T}_{i,period\_2} - \overline{T}_{i,period\_1} ,$$

3 $$\Delta P_{i} = \overline{P}_{i,period\_2} - \overline{P}_{i,period\_1} .$$

The relative difference (%) in period-averaged P (∆*P*_%,*i*_) was additionally calculated for each pixel as $\Delta P_{i} /\overline{P}_{period1} \times 100$, where $\overline{P}_{period1}$ is the average precipitation of period 1. If no precipitation was recorded in either of the periods (such as data value = 0, notably for arid regions), we considered the change to be equal to zero ((∆*P*_*i*_ = 0).

The zonal area-weighted averages of the hydro-climatic variables was estimated as,

4 $$\Delta T_{zone} = 1/n \cdot \sum\nolimits_{i \in zone} {w_{i} \Delta T_{i} } ,$$

5 $$\Delta P_{zone} = 1/n \cdot \sum\nolimits_{i \in zone} {w_{i} \Delta P_{i} } ,$$

where *zone* was set to either a delineated wetlandscape zone or a Köppen–Geiger climate zone, *w* is the weight factor of pixel *i* as defined previously, and *n* is the number of pixels within each zone. The zonal statistics of the climate zones are presented as “geographic box plots”, showing (in addition to the above defined average delta-changes; Eqs. (4) and (5)) the distribution of the weighted pixel data in the areas with minimum values (bottom whisker), first quartiles (bottom of box), medians (black line within the box), third quartiles (top of box) and maximum values (top whisker) (Figs. S1, S2). For graphical clarity, the main manuscript presents zoomed-in boxplots without whiskers (Figs. 1, 2). Since the considered arid, temperate and cold wetlandscapes are located in the northern hemisphere, we also calculated refined T and P trends for the respective shares of the climate zones that were located in the northern hemisphere.

Changes in T, P and R for all wetlandscapes were also statistically evaluated using the Wilcoxon Rank Sum test at a significance level of 95%. The Wilcoxon Rank sum test is a nonparametric test used to estimate differences between two samples, in this case the difference in T, P and R change between time periods 1 and 2 for each wetlandscape. Our null hypothesis was that there is no significant (p > 0.05) change between the two periods. The results of this test are presented in Table S2 found in the Supplementary Information.

## Supplementary Information

Supplementary Information.

## Data Availability

All data needed to evaluate the conclusions in the paper are present in the paper and/or the Supplementary Materials. The data of wetlandscapes from Russia was processed within RFBR project 18-05-60219 and for Volga River in particular—RFBR project 18-05-80094. Additional data related to this paper may be requested from the authors.
