# Publisher Correction: Hydro-climatic changes of wetlandscapes across the world

**DOI:** 10.1038/s41598-021-92697-9

**Published:** 2021-06-22

**Authors:** I. Åhlén, G. Vigouroux, G. Destouni, J. Pietroń, N. Ghajarnia, J. Anaya, J. Blanco, S. Borja, S. Chalov, K. P. Chun, N. Clerici, A. Desormeaux, P. Girard, O. Gorelits, A. Hansen, F. Jaramillo, Z. Kalantari, A. Labbaci, L. Licero-Villanueva, J. Livsey, G. Maneas, K. L. McCurley Pisarello, D. Moshir Pahani, S. Palomino-Ángel, R. Price, C. Ricaurte-Villota, L. Fernanda Ricaurte, V. H. Rivera-Monroy, A. Rodriguez, E. Rodriguez, J. Salgado, B. Sannel, S. Seifollahi-Aghmiuni, M. Simard, Y. Sjöberg, P. Terskii, J. Thorslund, D. A. Zamora, J. Jarsjö

**Affiliations:** 1 Department of Physical Geography and Bolin Center for Climate Research, Stockholm University, 10691 Stockholm, Sweden; 2 WSP Sverige AB, Ullevigatan 19, 411 40 Gothenburg, Sweden; 3 Facultad de Ingeniería, Universidad de Medellín, Carrera 87 30-65, 050026 Medellín, Colombia; 4 Facultad de Ciencias Exactas Y Naturales, Instituto de Biología, Universidad de Antioquia, Calle 70 No. 52-21, 050010 Medellín, Colombia; 5 Faculty of Geography, Lomonosov Moscow State University, Moscow, Russia 119991; 6 Department of Geography, Hong Kong Baptist University, SAR, Hong Kong, China; 7 Department of Biology, Faculty of Natural Sciences and Mathematics, Universidad del Rosario, 13409 Bogotá, DC Colombia; 8 School of Natural Resources and Environment, University of Florida, Gainesville, FL 32603 USA; 9 Centro de Pesquisa do Pantanal and BioScience Institute, Federal University of Mato Grosso, Cuiabá, Mato Grosso Brazil; 10 Zubov State Oceanographic Institute, Moscow, 119034 Russia; 11 Department of Civil, Environmental and Architectural Engineering, University of Kansas, Lawrence, KS 66045 USA; 12 Baltic Sea Centre, 10691 Stockholm, Sweden; 13 Department of Geology, Faculty of Sciences, Ibn Zohr University, Agadir, Morocco; 14 Institute of Botany and Landscape Ecology, University of Greifswald, 17489 Greifswald, Germany; 15 Navarino Environmental Observatory, 24 001 Messinia, Greece; 16 Department of Soil and Water Sciences, University of Florida, Gainesville, FL 32611 USA; 17 Facultad de Ingeniería, Universidad de San Buenaventura, Carrera 56C N° 51–110, 050010 Medellín, Colombia; 18 Department of Earth and Environment, Southeast Environmental Research Center, Florida International University, Miami, FL 33199 USA; 19 Instituto de investigaciones marinas y costeras de Colombia “José Benito Vives de Andreis”— INVEMAR, 470006 Santa Marta, Colombia; 20 Alexander von Humboldt Biological Resources Research Institute, Calle 28 A No. 15-09, 70803 Bogotá, DC Colombia; 21 Department of Oceanography and Coastal Sciences, College of the Coast and Environment, Louisiana State University, Baton Rouge, LA 70803 USA; 22 Civil and Agricultural Engineering Department, Universidad Nacional de Colombia, 11001 Bogotá, Colombia; 23 Departamento de Ciencias Biológicas, Universidad de Los Andes, Cra. 1 No. 18A-12, 111711 Bogotá, Colombia; 24 Facultad de Ingeniería, Universidad Católica de Colombia, Av. Caracas No. 46-72, 111311 Bogotá, Colombia; 25 Jet Propulsion Laboratory, California Institute of Technology, Pasadena, CA USA; 26 Department of Geosciences and Natural Resource Management, University of Copenhagen, Copenhagen, Denmark; 27 Department of Physical Geography, Utrecht University, Utrecht, The Netherlands

Correction to: *Scientific Reports* 10.1038/s41598-021-81137-3, published online 02 February 2021

In the original version of this Article, V. H. Rivera-Monroy was incorrectly affiliated with ‘Alexander von Humboldt Biological Resources Research Institute, Calle 28 A No. 15-09, Bogotá, DC, 70803, Colombia’. The correct affiliation is listed below.

Department of Oceanography and Coastal Sciences, College of the Coast and Environment, Louisiana State University, Baton Rouge, LA 70803, USA

As a result, Affiliations 22–27 were incorrectly listed as Affiliations 21–26 respectively.

The original Article has been corrected.

