# Supplementary material for: Hydro-climatic changes of wetlandscapes across the world
Source: Sci Rep. 2021 Feb 2;11:2754. doi: 10.1038/s41598-021-81137-3 (PMC7854620; doi:10.1038/s41598-021-81137-3)
Supplement: Supplementary file 1 — Supplementary Information. [file 41598_2021_81137_MOESM1_ESM.pdf]

Hydro-climatic changes of wetlandscapes across the world

Authors

I. Åhlén[1]*, G. Vigouroux[1], G. Destouni[1], J. Pietroń[1,2], N. Ghajarnia[1], J. Anaya[3], J. Blanco[4], S. Borja[1], S. Chalov[5], K.P. Chun[6], N. Clerici[7], A. Desormeaux[8], P. Girard[9], O. Gorelits[10], A. Hansen[11], F. Jaramillo[1,12], Z. Kalantari[1], A. Labbaci[13], L. Licero-Villanueva[14], J. Livsey[1], G. Maneas[1,15], K.L. McCurley Pisarello[16], D. Moshir Pahani[1], S. Palomino-Ángel[3 17], R. Price[18], C. Ricaurte-Villota[19], L. Fernanda Ricaurte[20], V.H Rivera-Monroy[21], A. Rodriguez[19], E. Rodriguez[22], J. Salgado [23,24], B. Sannel[1], S. Seifollahi-Aghmiuni[1], M. Simard [25], Y. Sjöberg[26], P. Terskii[5], J. Thorslund[1,27], D.A. Zamora [22], J. Jarsjö[1]

[1]Department of Physical Geography and Bolin Center for Climate Research, Stockholm University, 10691, Stockholm, Sweden
[2] WSP Sverige AB, Ullevigatan 19, 411 40 Gothenburg, Sweden
[3] Facultad de Ingeniería, Universidad de Medellín, Carrera 87 30–65, Medellín 050026, Colombia
[4] Instituto de Biología, Facultad de Ciencias Exactas y Naturales, Universidad de Antioquia, Calle 70 No. 52-21, Medellín 050010, Colombia
[5] Faculty of Geography, Lomonosov Moscow State University, Moscow 119991, Russia
[6] Department of Geography, Hong Kong Baptist University, Hong Kong SAR, China
[7] Department of Biology, Faculty of Natural Sciences and Mathematics, Universidad del Rosario, Bogotá 13409, DC, Colombia
[8] School of Natural Resources and Environment, University of Florida, Gainesville, Florida 32603, USA
[9] Centro de Pesquisa do Pantanal and BioScience Institute, Federal University of Mato Grosso, Cuiabá, Mato Grosso, Brazil
[10] Zubov State Oceanographic Institute, Moscow 119034, Russia
[11] Department of Civil, Environmental and Architectural Engineering, University of Kansas, Lawrence, Kansas 66045, USA
[12] Baltic Sea Centre, 10691, Stockholm, Sweden
[13] Department of Geology, Faculty of Sciences, Ibn Zohr University, Agadir, Morocco
[14] Institute of Botany and Landscape Ecology, University of Greifswald, Greifswald 17489, Germany
[15] Navarino Environmental Observatory, 24 001 Messinia, Greece
[16]Department of Soil and Water Sciences, University of Florida, Gainesville, Florida 32611, USA
[17]Facultad de Ingeniería, Universidad de San Buenaventura, Carrera 56C N° 51-110, Medellín 050010, Colombia
[18] Department of Earth and Environment, Southeast Environmental Research Center, Florida International University, Miami, Florida 33199, USA
[19]Instituto de investigaciones marinas y costeras de Colombia "José Benito Vives de Andreis"—INVEMAR, 470006 Santa Marta, Colombia
[20]Alexander von Humboldt Biological Resources Research Institute, Calle 28 A No. 15-09, Bogotá, D.C., Colombia
[21] Department of Oceanography and Coastal Sciences, College of the Coast and Environment, Louisiana State University, Baton Rouge, Louisiana 70803, USA
[22] Civil and Agricultural Engineering Department, Universidad Nacional de Colombia, Bogotá 11001, Colombia
[23] Departamento de Ciencias Biológicas, Universidad de Los Andes, Cra. 1 No. 18A-12, Bogotá 111711, Colombia
[24] Facultad de Ingeniería, Universidad Católica de Colombia, Av. Caracas No. 46-72, Bogotá 111311, Colombia
[25] Jet Propulsion Laboratory, California Institute of Technology, Pasadena, California, USA
[26] Department of Geosciences and Natural Resource Management, University of Copenhagen, Denmark
[27] Hydrology, Water resources, and Permafrost at the department, and at Utrecht University
[*] Corresponding author: imenne.ahlen@natgeo.su.se

Supplementary Materials

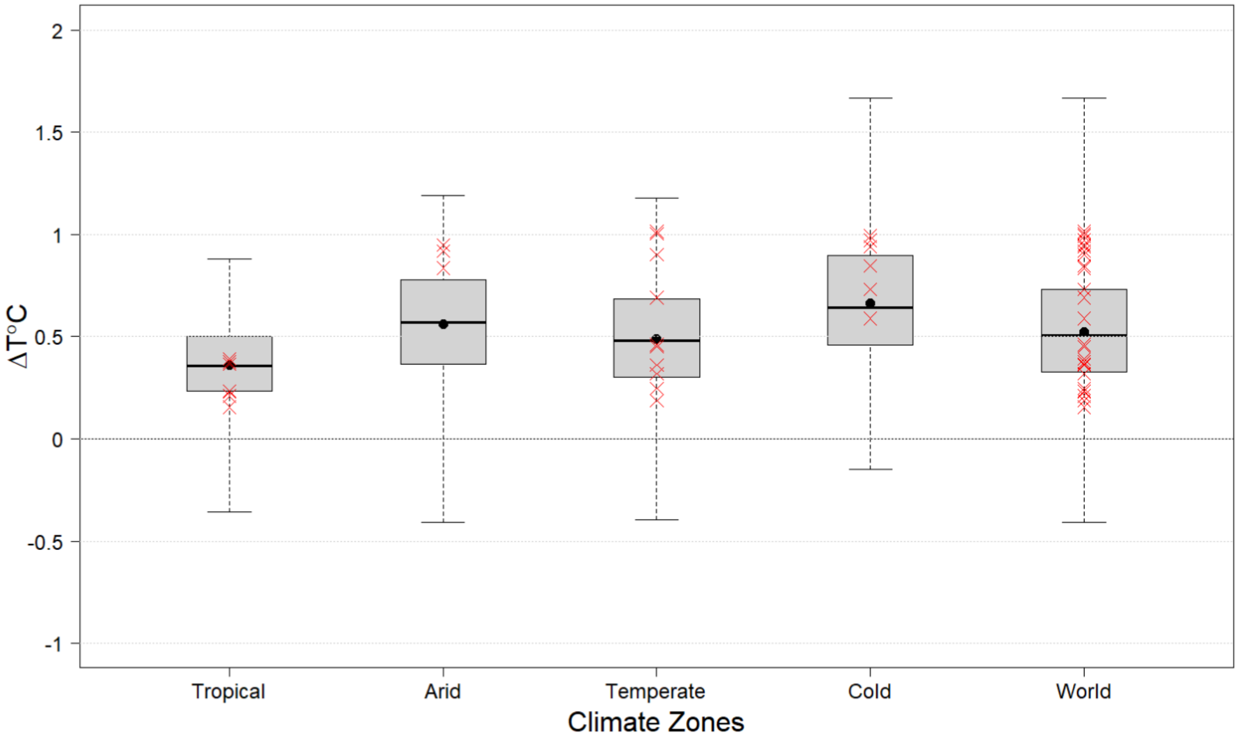

Fig. S1. Change in temperature ΔT°C. *Boxplots showing ΔT°C for each climate zone and wetlandscape between the period of 1976-1995 and 1996-2015 the mean ΔT°C for the climate zones are shown as black points and as red crosses for the wetlandscapes.*

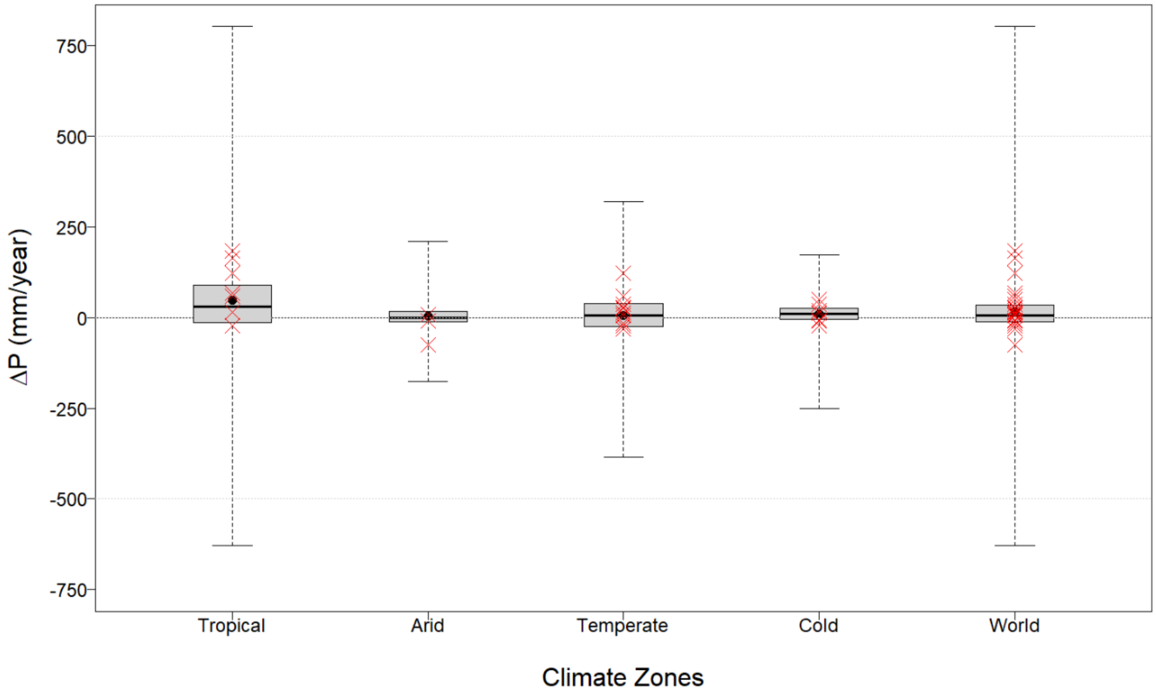

Fig. S2. Change in precipitation ΔP (mm/year). *Boxplot showing the ΔP for each climate zone and wetlandscape between the period of 1976-1995 and 1996-2015. Mean ΔP for the climate zones are shown as white points and as red crosses for the wetlandscapes*

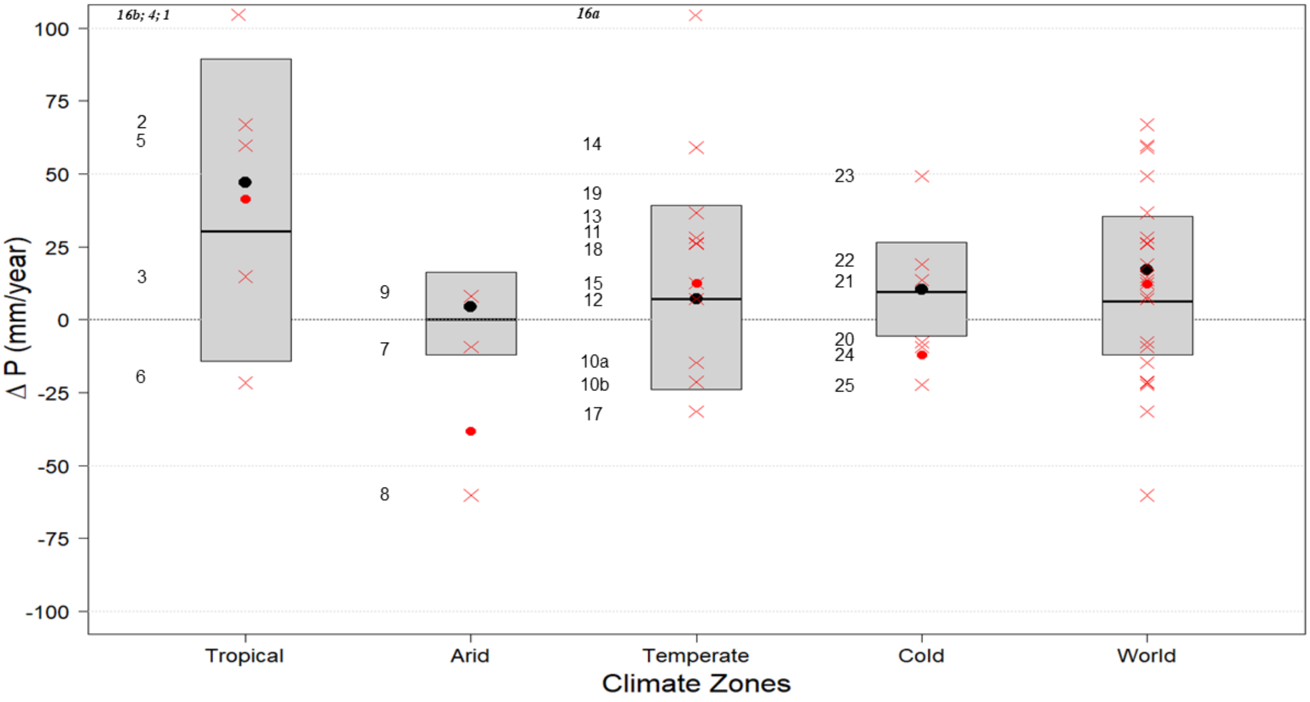

103 ***Fig. S3. Change in precipitation ΔP (mm/year).*** *Boxplot showing ΔP (mm/years) for each climate zone for the period between 1976-1995 and 1996-2015.* *The ΔP for each wetlandscape is presented as red crosses with a corresponding number relating to the name of the wetlandscape (see Table 1). Mean ΔP (mm/years) for the climate zones are shown as black points, while the red points show the mean ΔP (mm/years) for the wetlandscapes.*

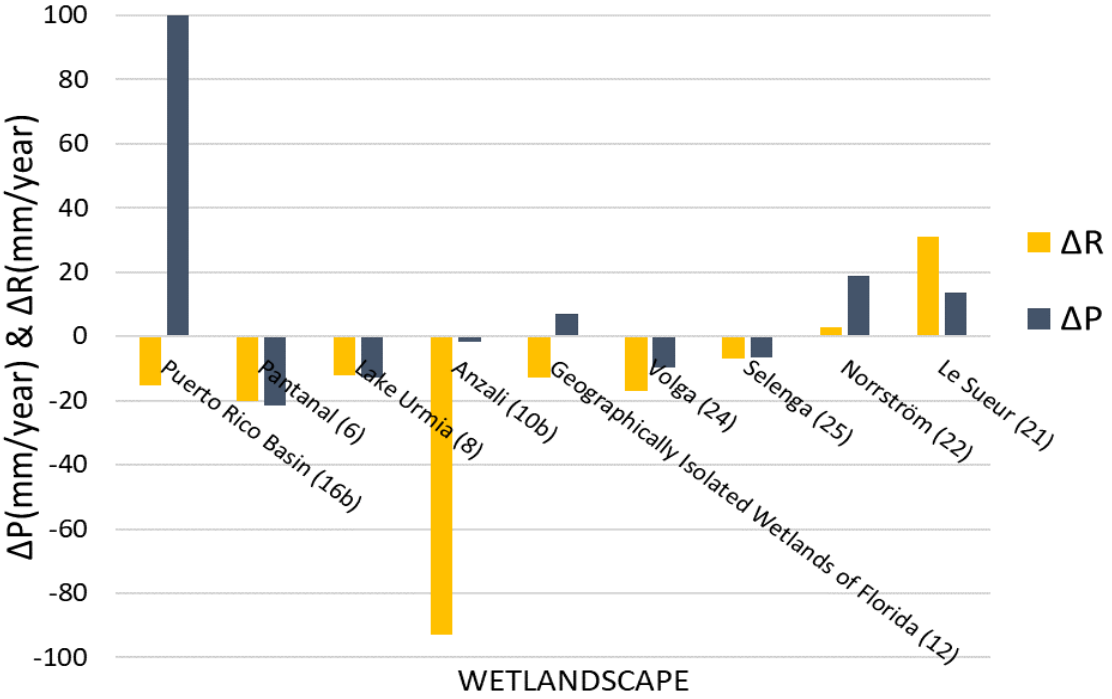

Fig. S4. Change in precipitation and runoff for the wetlandscapes with available discharge data. *The studied time period of change in precipitation and discharge are sometime differing between the wetlandscapes. This information can be read in Table 1 (found in manuscript).*

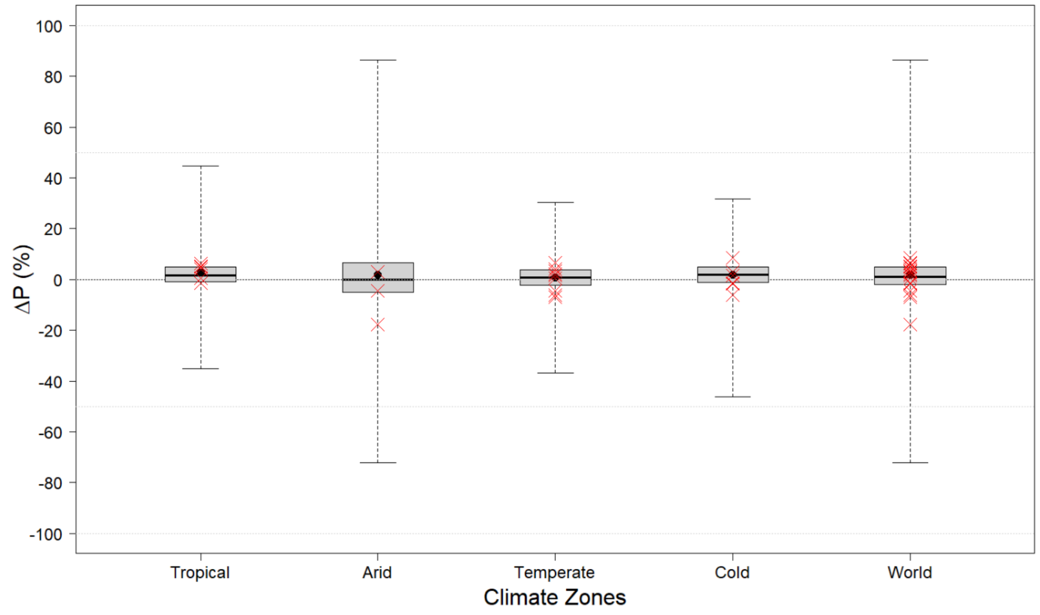

Fig. S5**. **Relative change in precipitation ΔP%. *Boxplot showing ΔP% for each climate zone and wetlandscape between the period of 1976-1995 and 1996-2015. Mean ΔP% for the climate zones are shown as white points and as red crosses for the wetlandscapes.*

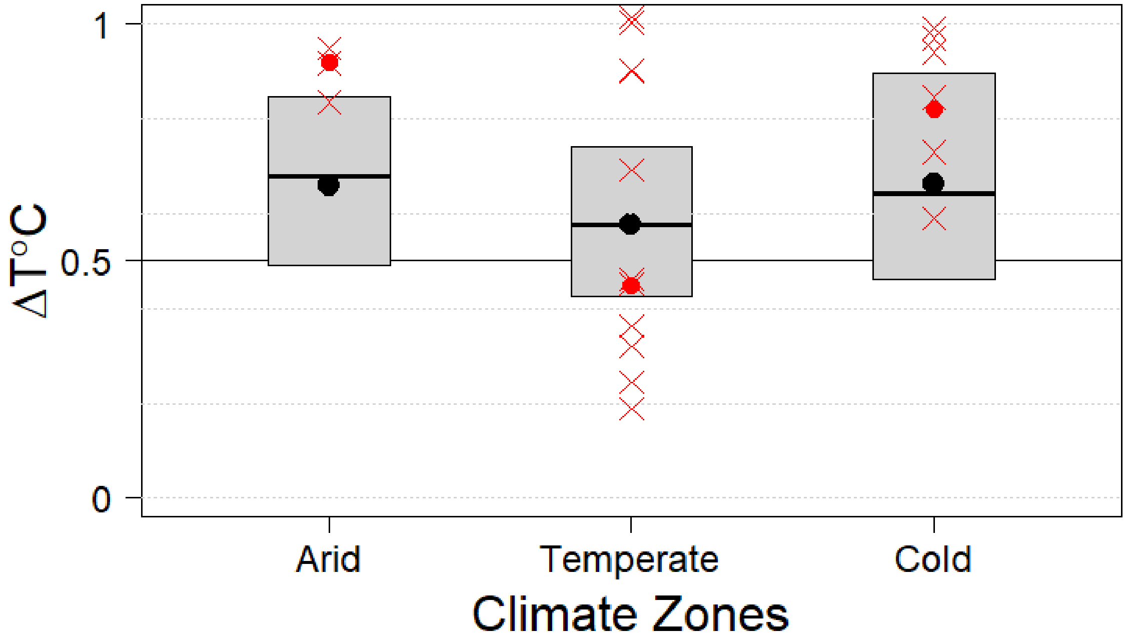

Fig. S6. Change in temperature (ΔT°C) for climate zone located in the north hemisphere. *Boxplot (without whisker) showing the ΔT°C for climate zone located in the north hemisphere between the periods of 1976-1995 and 1996-2015. The ΔT°C for each wetlandscape is shown as red crosses. Mean ΔT°C for the climate zones are shown as black points, while the red points show the mean ΔT°C for the wetlandscapes.*

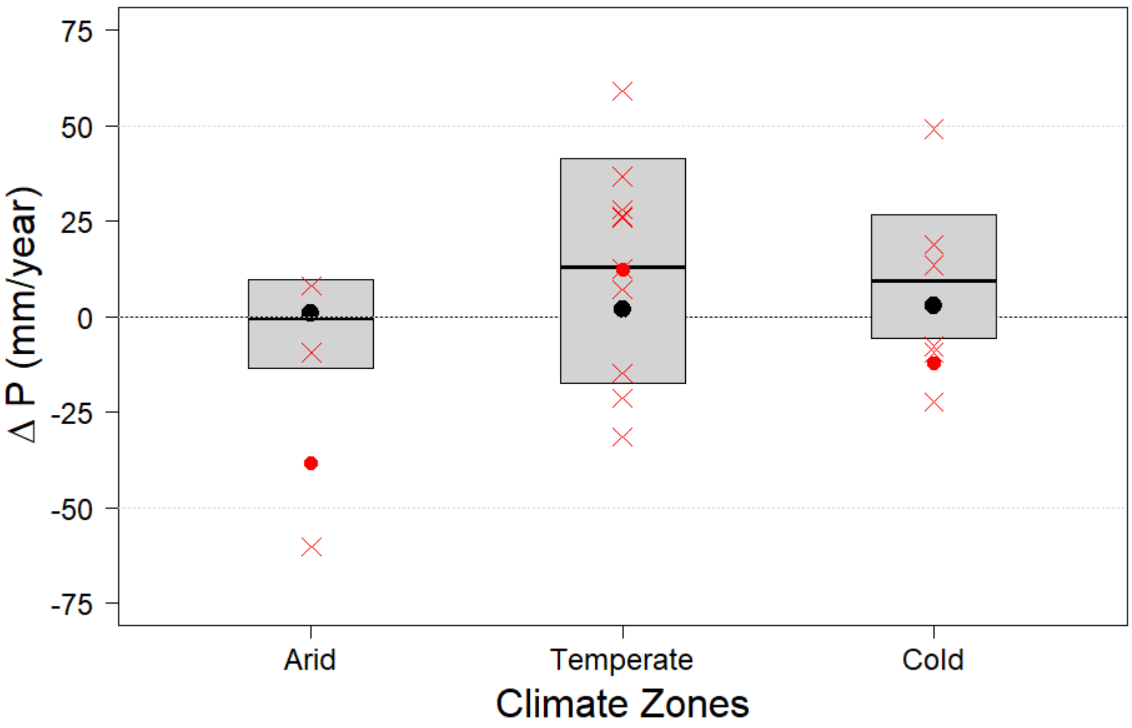

Fig. S7. Change in precipitation ΔP (mm/year) of climate zones located in the north hemisphere. *Boxplot showing ΔP (mm/years) for each climate zone located in the north hemisphere for the period between 1976-1995 and 1996-2015.* *The ΔP for each wetlandscape is presented as red crosses. Mean ΔP (mm/years) for the climate zones are shown as black points, while the red points show the mean ΔP (mm/years) for the wetlandscapes.*

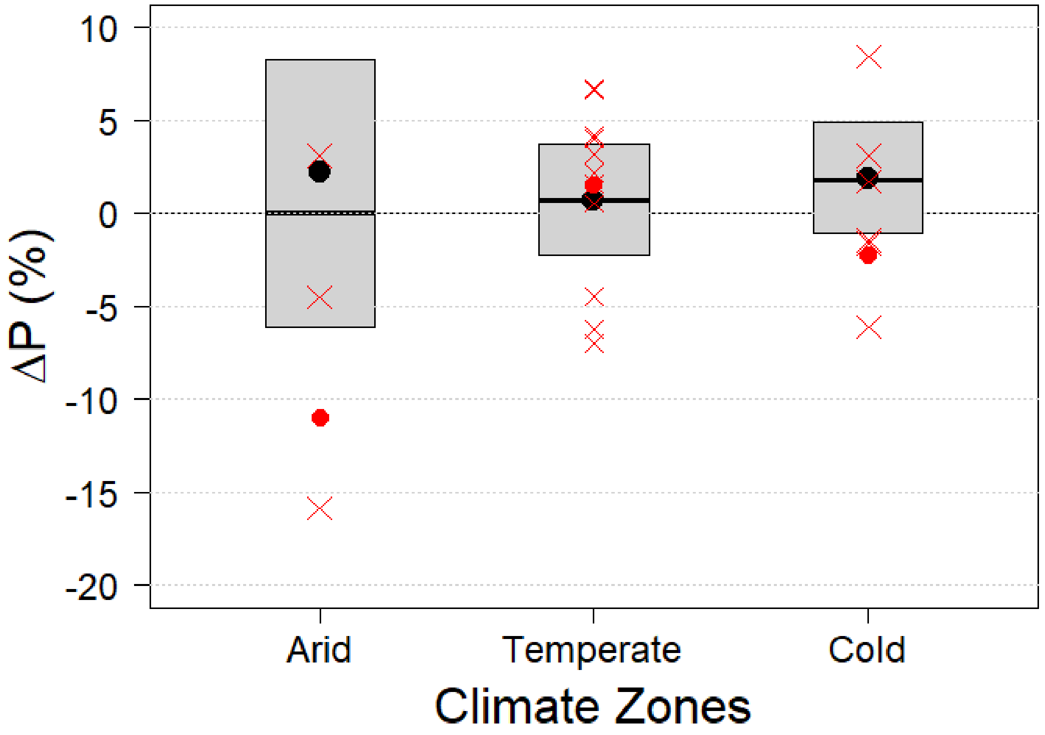

Fig. S8. Relative change in precipitation (ΔP%) of climate zones located in the north hemisphere. *Boxplots show the relative ΔP in percent for each climate zone located in the north hemisphere for the periods between 1976-1995 and 1996-2015. The relative ΔP for each wetlandscape is presented as red crosses. Mean ΔP% for the climate zones are shown as black points, while the red points show the mean ΔP% for the wetlandscapes. For detailed boxplots with whiskers, see supplementary material.*

Table S1. Characteristics of the wetlandscapes. *Table showing the characteristics of the wetlandscapes in terms of wetland type and wetland area coverage of the wetlandscapes.*

ID	Site name	Country	Wetland type	Area of wetlands relative to total catchment/wetland scape area (%)
1	Ciénaga Grande de Santa Marta	Colombia	Estuarine	0.77
2	Everglades	USA	Freshwater wetland, coastal wetland	32
3	Gatun Lake	Panama	Chagres River, lake	13
4	León–Atrato	Colombia	Marshes and swamps	17
5	Mekong	Vietnam	Marine	5
6	Pantanal	Brazil	Periodically inundated savanna	27
7	Shadegan	Iran	Palustrine, estuarine, marine	31
8	Lake Urmia	Iran	Lake	8.8
9	Zone Humide de Souss	Morocco	Marine and coastal	0.01
10	Anzali Mordab	Iran	Inland and marine/coastal wetland	4
11	Gialova Lagoon	Greece	Coastal wetland	13
12	Geographically isolated wetlands	USA	Freshwater marshes and swamps	30
13	Lagunas Plaza and Grande	Colombia	Glacial lake	4.4
14	Fúquene, Cucunubá y Palacio	Colombia	Natural shallow lake	1.7
15	Lower Mississippi River Delta Plain	USA	Riverine, marine, estuarine, Lacustrine	3.5
16	Páramo Sumapaz	Colombia	High-altitude wetland	46
17	Sacca Di Goro	Italy	Shallow saltwater coastal lagoon	4.2
18	Simpevarp	Sweden	Bogs, fens	0.01
19	Upper Lough Erne	Ireland	Flood plain/shallow lakes	22
20	Forsmark	Sweden	Bogs, fens, marshes, (shallow lakes)	0.01
21	Le Sueur	USA	Isolated, fluvial/riparian, lakes/ponds, marshes, forest/shrubs, constructed	5
22	Norrström	Sweden	Multiple	5
23	Tavvavuoma	Sweden	Peat plateau/thermokarst lake complex	2.8
24	Volga	Russia	Marshes (riverine, palustrine)	1
25	Selenga	Russia	Marshes (riverine, palustrine)	0.13

Table S2. Results of the Wilcoxon Rank Sum test at 95% confidence level*. Table showing resulting p-values from the Wilcoxon Rank Sum test at 95 % confidence level between two sample periods (period 1 and period 2) for precipitation (P), temperature (T) and runoff (R) for each wetlandscape. Significant results (p < 0.05) are shown in bold.*

ID	***Wetlandscapes***	***p-value (P)***	***p-value (T)***	***p-value (R)***
1	*Ciénaga Grande de Santa Marta*	0.232	**0.038**	
2	*Everglades*	0.149	**0.013**	
3	*Gatun Lake*	0.529	**0.009**	
4	*León-Atrato*	0.301	**0.046**	
5	*Mekong*	0.097	**0.001**	
6	*Pantanal*	0.478	**0.000**	**0.00004**
7	*Shadegan*	0.820	**0.000**	
8	*Lake Urmia*	**0.006**	**0.000**	0.217
9	*Zone Humide de Souss*	0.989	**0.000**	
10	*Anzali Mordab*	0.183	**0.000**	**0.020**
11	*Gialova Lagoon*	0.862	**0.000**	
12	*Geographically Isolated Wetlands of Florida*	0.639	**0.049**	0.221
13	*Lagunas Plaza and Grande*	0.583	**0.002**	
14	*Fúquene, Cucunúba Y Palacio*	0.201	**0.023**	
15	*Lower Mississippi River Delta Plain*	0.968	**0.043**	
16	*Páramo Sumapaz*	0.232	**0.060**	0.463
17	*Sacca Di goro*	0.301	**0.000**	
18	*Simpevarp*	0.242	**0.001**	
19	*Upper Lough Erne*	0.398	**0.000**	
20	*Forsmark*	0.718	**0.001**	
21	*Le Sueur*	0.947	**0.043**	0.429
22	*Norrström*	0.800	**0.001**	0.883
23	*Tavvavuoma*	0.091	**0.002**	
24	*Volga*	0.904	**0.012**	0.091
25	*Selenga*	0.149	**0.002**	0.274
